# Efficient Film Fabrication and Characterization of Poly(3,4-ethylenedioxythiophene):Poly(styrenesulfonate) (PEDOT:PSS)-Metalloporphine Nanocomposite and Its Application as Semiconductor Material

**DOI:** 10.3390/polym13224008

**Published:** 2021-11-19

**Authors:** María Elena Sánchez-Vergara, Leon Hamui, Daniela González-Verdugo, Ismael Cosme

**Affiliations:** 1 Facultad de Ingeniería, Universidad Anáhuac México, Avenida Universidad Anáhuac 46, Colonia Lomas Anáhuac, Huixquilucan 52786, Estado de México, Mexico; elena.sanchez@anahuac.mx (M.E.S.-V.); daniela.gonzalezve@anahuac.mx (D.G.-V.); 2 Investigador por México CONACYT-INAOE, National Institute of Astrophysics, Optics and Electronics (INAOE), Luis Enrique Erro #1, Tonantzintla 72840, Puebla, Mexico; ismaelcb@inaoep.mx

**Keywords:** PEDOT:PSS, porphine, composite film, vapor treatment, electrical properties

## Abstract

The use of composite films with semiconductor behavior is an alternative to enhance the efficiency of optoelectronic devices. Composite films of poly(3,4-ethylenedioxythiophene):poly(styrenesulfonate) (PEDOT:PSS) and metalloporphines (MPs; M = Co, Cu, Pd) have been prepared by spin-coating. The PEDOT:PSS-MP films were treated with isopropanol (IPA) vapor to modify the polymer structure from benzoid to quinoid. The quinoid structure promotes improvements in the optical and electrical behavior of films. The composite films’ morphology and structure were characterized using infrared and Raman spectroscopy, scanning electron microscopy (SEM), and atomic force microscopy (AFM). Composite films were analyzed for their optical behavior by ultraviolet-visible spectroscopy: at λ < 450 nm, the films become transparent, indicating the capacity to be used as transparent electrodes in optoelectronic devices. At λ ≥ 450 nm, the absorbance in the films increased significantly. The CoP showed an 8 times larger current density compared to the CuP. A light induced change in the J-V curves was observed, and it is larger for the CoP. The conductivity values yielded between 1.23 × 10^2^ and 1.92 × 10^3^ Scm^−1^ and were higher in forward bias.

## 1. Introduction

**Figure 1:**
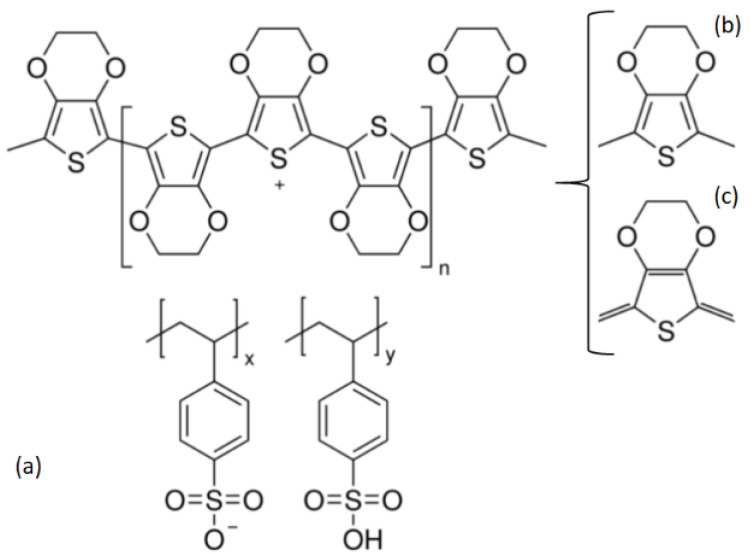
Structure of (**a**) PEDOT:PSS. (**b**) Benzoid and (**c**) quinoid structures of PEDOT.

**Figure 2:**
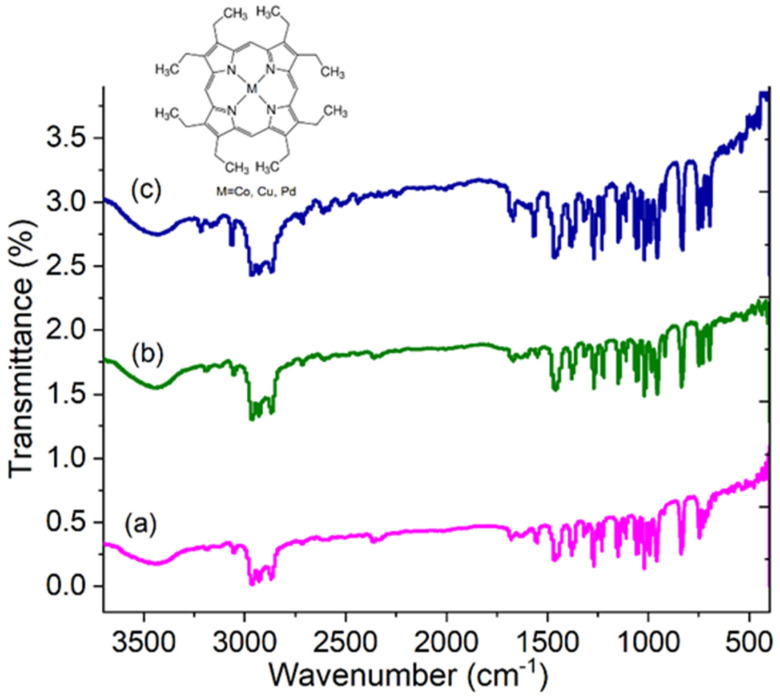
FTIR spectra of (**a**) CoP, (**b**) CuP, and (**c**) PdP in KBr pellets.

Organic devices could be competitive for low-cost applications in electronics requiring structural flexibility and low temperature processing [1]. During the last decades, much effort has been put into the development of organic devices based on conjugated polymers and small molecules [2]. The considerable activity has been spurred by the simple fabrication of large-area flexible thin film devices [2]. Thin films of conjugated polymers and small molecules are versatile and widely investigated for their use as chemical sensors [3], information storage [4], non-linear optical material [5], and optoelectronic devices [6,7,8,9]. The main characteristic of conjugated polymers is the alternation of the single and double bonds between the carbon atoms along the chain. The electronic properties of these polymer semiconductors are related to their extended conjugation, in which the σ electrons are delocalized and are easily polarizable, so they can move along the chain and transmit electronic charges [10,11]. Compared to conjugated polymers, small molecules possess defined molecular structure and molecular weight for convenient synthesis, which improves the fabrication repeatability, as well as exhibiting a greater tendency to form ordered domains, affording higher charge carrier mobilities [12]. Small molecules and polymeric conductors comprise an extended carbon-based π-conjugated structure [13]. The π-conjugated system forms a delocalized cloud of electrons with an alternating periodic density, which generates conduction channels for electric charges flow. However, there are many material selection criteria to consider in developing organic devices including, but not limited to, π-electron richness, aromaticity, electrical conductivity, dipole moments, frontier energy levels, thermal stability, and chemical stability [14]. Poly(3,4-ethylenedioxythiophene):poly(styrenesulfonate) (PEDOT:PSS), shown in in Figure 1a, proved to meet these requirements well and is the most widely used transparent electrode and hole transport layer (HTL) in standard geometry bulk heterojunction (BHJ) solar cells [15,16]. PEDOT:PSS has exhibited outstanding optical transparency in the visible region, solution processability, high work function value (5.1 eV), excellent mechanical tensility, and high thermal stability [16,17,18,19]. However, despite possessing very important qualities, the PEDOT:PSS in film form does not have sufficient electrical conductivity compared to other polymers such as the polyphenylenevinylenes [12]. This issue has been solved by various means, among which the addition of secondary dopants for the manufacture of composite films stands out. Some PEDOT:PSS in composite films application examples are reported by Kepić et al. [20] that use spherical nanoparticles of graphene embedded in the PEDOT:PSS, and by Sarkhan et al. [21] with graphene oxide sheets embedded in the polymer. In contrast; Stevens et al. [22] carried out the improvement of thermoelectric performance of polymer nanocomposites, and also Yeo et al. [23] carried out an improvement in the electrical behavior of composite films with PEDOT:PSS. It is important to consider that the study of the secondary dopants in the PEDOT:PSS has not been extended to the use of small molecules such as porphines (Figure 2). Porphines are discotic systems that allow the transport of charges in a single direction, generated by: (i) the presence of the central metal ion, through which the conduction channel is formed [24], and (ii) its capacity to be organized in practically one-dimensional columns [24,25]. The porphine is an aromatic heterocyclic compound with a 12-carbon outside ring and four embedded pyrrole rings. The packing for porphine was considered rare for compounds of the same dimensions, because porphine exhibits no close inter-molecular contact [24]. Fischer reported the first synthesis of porphine by adding pyrrole-α-aldehyde to boiling formic acid [25], and the spectroscopic results were confirmed by Rothemund [26]. Porphine is the parent compound of the porphyrins series [24] and contains a metal atom coordinated to the four nitrogen atoms. Porphine is the simplest porphyrin and represents the core macrocycle of naturally occurring and synthetic porphyrins [27]. The potential of this compound is enormous and it could be advantageous to use the porphine unit as a building block for optoelectronic applications because of its planar structure, its conjugated bonds, and its high density of delocalized π-electrons. Theoretical studies have targeted the interaction of porphine and its metal complexes with fullerenes. Fullerenes bounded to porphine may be used in data storage media, photovoltaic and electrochemical devices, and gas sensors [24]. However, despite first being synthesized over 90 years ago, porphine has not been utilized to its full extent due to low yield syntheses and expense [24]. Additionally, the apolar tetrapyrrolic ring structure of porphine means it is poorly soluble in most organic solvents [24,28]. Currently, the development of organic devices integrated by solid-state films opens an important field of study for these compounds. The originality of this work focuses in the optoelectronic study of the potential use of porphines as a secondary dopant and their solid-state devices. It is because of the above that the present work is a systematic study on the effect of porphine on optoelectronic characteristics of PEDOT:PSS-porphine composite films made by spin-coating. It is important to note that porphines of manganese (MnP), iron (FeP), and zinc (ZnP) are the metalloporphines (MPs) that have been studied mainly as thin films [29,30,31,32]. Therefore, the current work extends the study of the MPs to the cobalt (CoP), copper (CuP), and palladium (PdP) porphines in order to know the effect of metal in the porphine and its function inside the PEDOT:PSS composite films. The aim in this work is to fabricate highly efficient composite films by exploiting the benefits of the enhanced electrical conductivity of the PEDOT:PSS. The composite films were post-treated by isopropanol (IPA) vapor exposure in order to improve the electronic behavior of the films by changing the structure of the polymer from benzoid to quinoid (Figure 1b). The optical behavior of composite films was investigated before and after the IPA treatment. Finally, the electrical characterization of the PEDOT:PSS-porphine films made it possible to establish their possible applications in electronic devices.

## 2. Materials and Methods

The materials poly(3,4-ethylenedioxythiophene)-poly(styrenesulfonate) (PEDOT:PSS), 2,3,7,8,12,13,17,18-Octaethyl-21*H*,23*H*-porphine cobalt(II) (CoP), 2,3,7,8,12,13,17,18-Octaethyl-21H,23H-porphine copper(II) (CuP), and 2,3,7,8,12,13,17,18-Octaethyl-21H,23H-porphine palladium(II) (PdP) were obtained from commercial suppliers (Sigma-Aldrich, Saint Louis, MO, USA) and used without further purification. In the composite films fabricated by spin-coating technique, a Smart Coater 200 (Laurell Technologies Corporation, North Wales, PA, USA) was used. The dispersion used for the manufacture of the films consisted of 6 mL of PEDOT:PSS and MP (M = Co, Cu, Pd) from a dilution of 2.8 wt% in H_2_O. The mixture was dispersed using the G560 shaker of Scientific Industries Vortex-Genie (Bohemia, NY, USA). The dispersion was later deposited on the substrate and the equipment was operated for one time at an angular speed of 800 rpm for 8 s spin time and a 250 rpm/s acceleration. In this experiment, 5 drops of dispersion were added onto the substrate with a plastic syringe before each spin, then the spin-coating equipment was started and after a 3 min drying at 80 °C, this process was repeated 3 times. Finally, the films were post-treated by vapor exposure of isopropanol (IPA) heated at 40 °C for 10 min. Composite films were deposited in silicon wafer (single side polished) n-type and p-type, Corning glass and indium tin oxide (In_2_O_3_·(SnO_2_)_x_; ITO) coated glass slide. The Corning glass and the glass-ITO substrates were at first sequentially washed in an ultrasonic bath with dichloromethane, methanol, and acetone. The silicon substrates were washed with a solution of 10 mL HF, 15 mL HNO_3_, and 300 mL H_2_O. FTIR spectroscopy analysis was performed using a Nicolet iS5-FT spectrometer (Thermo Fisher Scientific Inc., Waltham, MA, USA) at a wavelength range of 4000 to 400 cm^−1^. Infrared spectroscopy was performed in order to monitor the main functional groups of the porphines and verify that after depositing the composite films, no decomposition of the porphines has occurred. Raman spectra were measured using an AFM-Raman of Ntegra Spectra systems from NT-MDT Inc. (Liestal, Switzerland) with a wavelength of the excitation laser of 532 nm. Raman spectroscopy was performed mainly to verify the change of PEDOT from its benzoid structure to its quinoid structure. Topographic characteristics of films and their roughness were investigated in contact mode with a Nanosurf Naio (Nanosurf, Liestal, Switzerland) atomic force microscope (AFM), and morphologic characteristics in composite films were investigated with a Hitachi SU3500 (Hitachi, Tokyo, Japan) scanning electron microscope (SEM). The UV-vis spectra were obtained in the 200–1100 nm wavelength range with a UV-Vis 300 Unicam spectrophotometer UV300 (Thermo Fisher Scientific Inc., Waltham, MA, USA). The UV-vis spectroscopy was performed in order to obtain the optical parameters of the composite films such as transmittance, absorbance, and optical bandgap. For the electrical characterization, simple devices were fabricated using ITO as anode and silver as cathode. A programmable voltage source, a sensing station with lighting and temperature controller circuit from Next Robotix (Comercializadora KMox, S.A. de C.V., Mexico City, Mexico), and an auto-ranging Keithley 4200-SCS-PK1 pico-ammeter (Tektronix Inc., Beaverton, OR, USA) were employed.

## 3. Results and Discussion

### 3.1. Structural and Morphological Characterization

Initially the IR spectroscopy measurements of the porphines were performed with the purpose to identify their main bonds. Subsequently, during the deposit of the films with PEDOT:PSS, the measurements could monitor their chemical stability. The intensity and frequency of the absorbed IR are strongly dependent on the type of the bond. FTIR spectra of powder porphines are illustrated in Figure 2 and the observed IR bands and their assignments are presented in Table 1, being in a good agreement with the literature [29,30,33,34,35,36]. The spectra of the three metalloporphines (MPs M = Co, Cu, Pd) are almost similar except for some minor differences that appear in the relative intensity, shape, and position of some bands [30].

**Figure 3:**
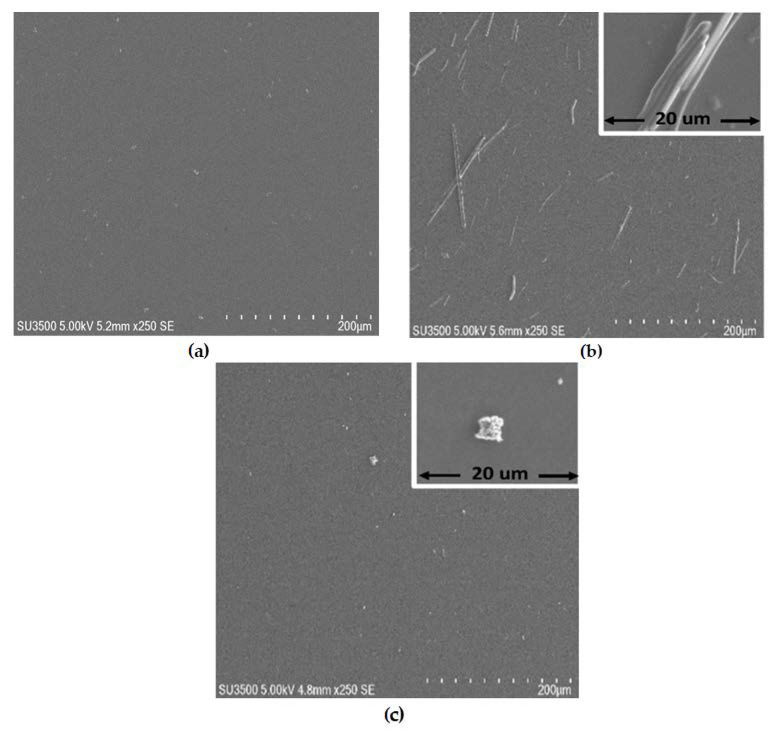
The SEM micrographs (**a**) PEDOT:PSS-CoP film; (**b**) PEDOT:PSS-CuP film and long-bar microstructure; (**c**) PEDOT:PSS-PdP film and cluster microstructure.

**Figure 4:**
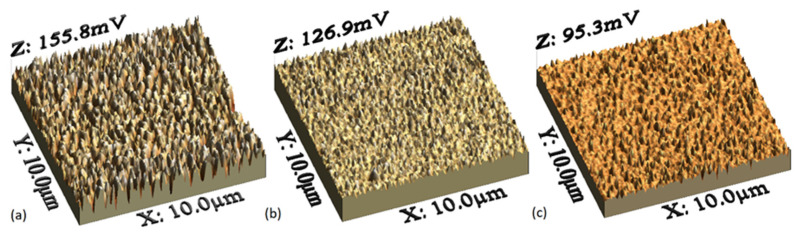
AFM images of (**a**) PEDOT:PSS-CoP, (**b**) PEDOT:PSS-CuP, and (**c**) PEDOT:PSS-PdP composites films.

Films composed by MPs and the PEDOT:PSS used as matrix were deposited by spin-coating. After the preparation of the films, they were treated with isopropanol (IPA) steam, in order to transform PEDOT:PSS from its benzoid to quinoid form (see Figure 1) [29,30,31,32,33,34,35,36,37,38,39]. The benzoid structure is present in its base state, whereas the quinoid structure is dominant in the doped state [40]. The morphology of films was investigated using the SEM and AFM. Figure 3a–c show the SEM images of the (a) PEDOT:PSS-CoP, (b) PEDOT:PSS-CuP, and (c) PEDOT:PSS-PdP composites films. The films show a very smooth, flat, and homogeneous surface, demonstrating the uniform incorporation of the metalloporphines to the bulk of the PEDOT:PSS matrix. These also show the presence of waste microparticles on the surface. It is important to note that the shape of the micro structures presented on the surface can be associated with the film composition: spherical (Figure 3c: inset) for the Co [41] and Pd [42] and long bars (Figure 3b: inset) for Cu composition [43]. It is expected the formation of similar micro grains dominating the bulk of the composite films. The creation of micro grains is the better pathway for the transportation of charge carriers in the polymer matrix [44]. The 3D AFM images of composite films are depicted in Figure 4, and they are studied to observe the surface roughness (see Table 2) and morphological features of the deposited films. The composite films show significant changes in morphology with the presence of each metalloporphine. The inclusion of the porphine in PEDOT:PSS reduces the segregation between PEDOT and PSS, with a reduction in electrostatic force of attraction between the conducting PEDOT and insulating PSS [44]. In PEDOT:PSS-CoP films (Figure 4a) the root mean square (RMS) roughness is the largest for the three films. This RMS indicates that the CoP particles tend to join together where grain growth occurs. The CoP interacts with PEDOT and PSS chains, and a denser morphology is formed in PEDOT:PSS films. In the case of the PEDOT:PSS-CuP films (Figure 4b), the roughness decreases considerably with respect to the film with the CoP particles, although its value is also high, due to the presence of the long bars structures that are clearly observed in Figure 3b. Finally, the PEDOT:PSS-PdP films (Figure 4c) present the lowest roughness. The control of the morphological characteristic of these films should be deeply studied in futures work depending on the specific application. For example, rough surfaces can improve the light absorption in solar cell structures [27,40]; however, applications such as diodes or organic transistors require very low roughness. It was demonstrated that higher solution concentrations in the preparation and deposition process of pristine PEDOT:PSS films resulted in more uniform films and higher spinning speed results in better phase segregation [45].

**Figure 5:**
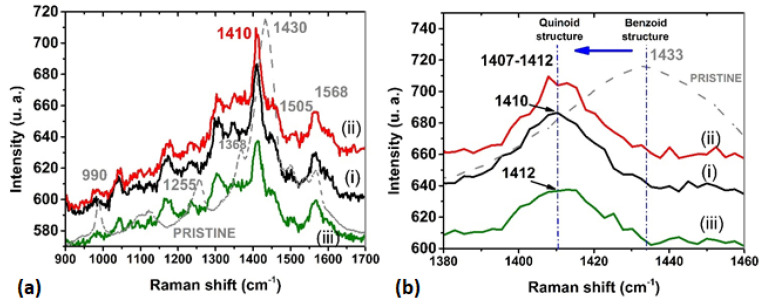
(**a**) Raman spectra of (i) PEDOT:PSS-CoP, (ii) PEDOT:PSS-CuP, (iii) PEDOT:PSS-PdP composite films and PEDOT:PSS pristine; (**b**) Close view of Raman associated to C=C symmetrical stretching intramolecular vibration (1400–1440 cm^−1^) and shift from benzoid to quinoid structure.

Figure 5 shows the Raman spectra for the films composed of (i) PEDOT:PSS-CoP, (ii) PEDOT:PSS-CuP, (iii) PEDOT:PSS-PdP, and PEDOT:PSS pristine. Raman spectroscopy is a sensitive technique to investigate conformational changes on the PEDOT:PSS films. Figure 5a presents the Raman spectrum of the pristine PEDOT:PSS film as reference and the main peaks of the chain conformation bands are identified as follows [37,38,39,40,44,46,47,48]: oxyethylene ring deformation at 990 cm^−1^, C_α_–C_α_ inter-ring stretching at 1255 cm^−1^, the C_β_–C_β_ stretching mode at 1368 cm^−1^, C_α_=C_β_ symmetric stretching vibrations at 1430 cm^−1^, C_α_=C_∞_ asymmetrical stretching at 1505 cm^−1^, and the C=C asymmetric stretching vibration at 1568 cm^−1^. It is observed that in comparison to pristine PEDOT:PSS films, the composite films presents a change in the peak distribution of their Raman spectra. The changes are manly observed in the peaks of the C_α_–C_α_ inter-ring stretching, the C_β_–C_β_ stretching mode, and the C_α_=C_β_ symmetric stretching vibrations of the five-membered thiophene ring on PEDOT that occur between 1400 and 1500 cm^−1^. In contrast, the asymmetrical stretching bands, 2505 and 1568 cm^−1^, remain their position peak in the MPs composites films in reference to the pristine PEDOT:PSS film. Studies on PEDOT Raman spectra demonstrate that changes in the C=C stretching vibration are evidence of a change of resonance in the PEDOT chains from benzoid to a quinoid form. Figure 5b analyzes the position of these band between 1400 and 1440 cm^−1^ for the pristine and MPs composite films. The composite films show a large shift in the C_α_=C_β_ stretching vibration peak position from the reference 1430 cm^−1^ to red, PdP (~1412 cm^−1^), CoP (~1410 cm^−1^), and CuP (~1407–1412 cm^−1^), meaning a change from the benzoid to the quinoid structure. A deformation of the spectra in comparison to the pristine in the 1100–1410 cm^−1^ region is also observed. The changes in the MPs film spectra can be attributed to the pairs of π-electrons in conjugation in the macrocycle ring involving the C–C and the C=C bonds, and the C–N bonds of the pyrrole rings. Among the most characteristic peaks that porphines incorporate that results in the deformation of the spectra are the signal around 1488 cm^−1^ (ν C=C Ph), 1383 cm^−1^, 1371 cm^−1^, 1355 cm^−1^, and 1120 cm^−1^ (ν C–N), the signal at 1095 cm^−1^ (δ C–H Ph), and the peak at 990 cm^−1^ (C–C) [49]. This indicates the successful incorporation of porphines to the PEDOT:PSS matrix and that the metalloporphines did not suffer degradation during the deposition process; this demonstrates that the spin-coating technique is suitable for the manufacture of composite films.

**Figure 6:**
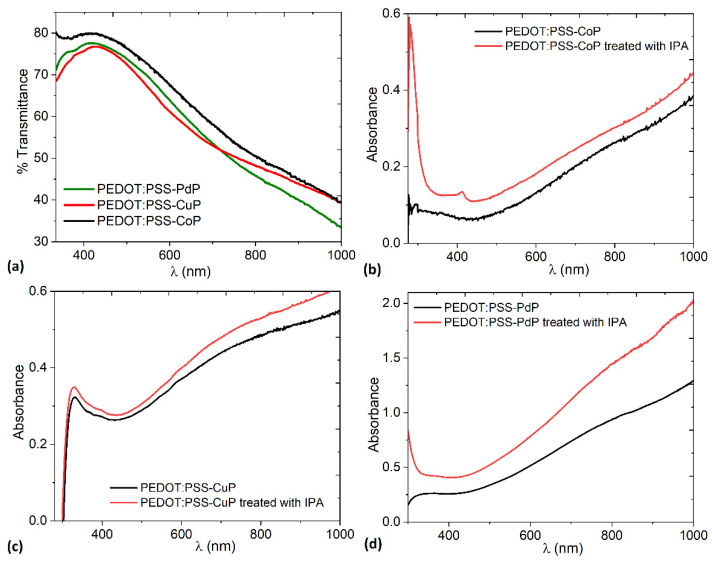
Spectral behavior of the (**a**) transmittance and absorbance for (**b**) PEDOT:PSS-CoP, (**c**) PEDOT:PSS-CuP, and (**d**) PEDOT:PSS-PdP films without and with IPA treatment.

The transmittance of the composite films as a function of the wavelength is shown in Figure 6a. The three films have practically the same behavior. The spectra can be divided into two regions: (a) in the spectral region where λ < 450 nm, the transmittance was detected to be relatively high with a value approximately 78%. Apparently, at λ < 450 nm all films become transparent, indicating that there is no light absorbed by a non-absorbing region [29,30]. These results are an indication of the capacity of the composite films to be used as transparent electrodes in optoelectronic devices [30,50]. (b) At λ ≥ 450 nm, the transmittance decreases considerably, and in Figure 6b–d it is observed how the absorbance increases. After treatment with IPA, the absorbance in the films increases even further because of the change in structure of the PEDOT. Additionally, it is important to consider that all the films were manufactured under the same conditions, in such a way that the only difference between them is the metal ion in the porphine. The highest absorbance occurs in the film with PdP, probably due to the larger size of the palladium ion with respect to cobalt and copper ions. This larger ion size generates a distortion in the macrocycle, which favors the transport of charges between the orbitals of the porphine [32,51].

**Figure 7:**
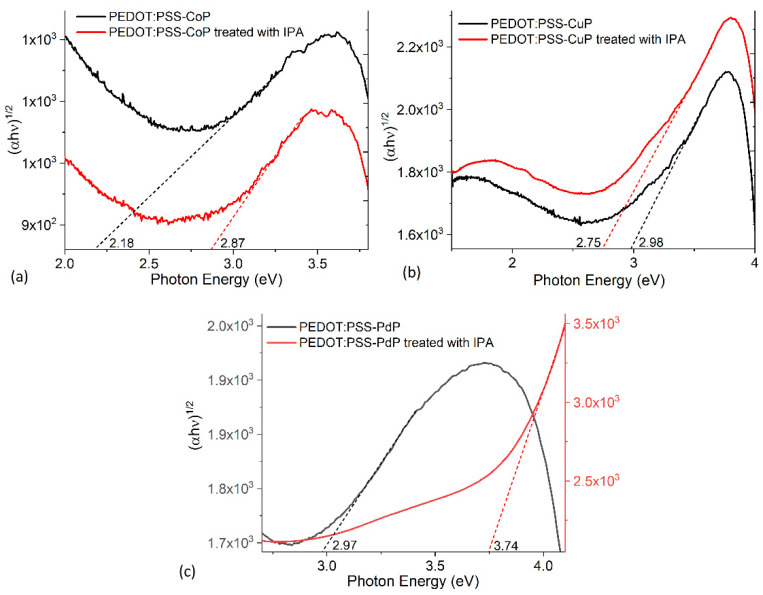
Bandgap plots for (**a**) PEDOT:PSS-CoP, (**b**) PEDOT:PSS-CuP, and (**c**) PEDOT:PSS-PdP films without and with IPA treatment.

The bandgap of semiconductor films is an important parameter in designing optoelectronic devices. Bardeen et al. [30,52] related the band gap of films to its absorption coefficient (α) and incident photon energy (hν) as: (αhν) = B(hυ−E_g_)^r^, where E_g_ is the bandgap energy, B depends on the type of transition, and r is the power that can equal 2 for indirect electronic transitions [30]. The bandgap is determined by plotting (αhν)^1/2^ versus hυ and finding the intercept on the hυ axis by extrapolating the plot to (αhν)^1/2^ = 0. Figure 7 and Table 2 show the bandgaps obtained for PEDOT:PSS-MP films before and after the IPA treatment. It is observed that in PEDOT:PSS-CuP the treatment generated a decrease in the bandgap as a result of the transformation in the polymer structure. According the Table 2, the values of optical energy bandgap are approximately 2.87 eV for PEDOT:PSS-CoP films, 2.75 eV for PEDOT:PSS-CuP films, and 3.74 eV for PEDOT:PSS-PdP films. For this latest composite film, the increase in bandgap is considerable and apparently due to the change in polymer structure and the presence of the palladium ion with the largest size compared to the cobalt and copper ions. This larger size in the metal distorts the porphine macrocycle, reducing charge transport both intermolecularly and intramolecularly. The bandgap results are higher than those obtained for porphine iron films (1.63 eV [31]), and for porphine manganese films (2.48 eV [30], 1.81, and 2.41 eV [29]). However; in all cases for the PEDOT:PSS-MPs, the bandgap is within the interval required for semiconductor films [13] and, apparently, the dispersed heterojunction that is formed from the polymer and the porphine allows the interconnection between both species and the transport of charges through them [2]. To determine the possible use of films in optoelectronic devices, it is necessary to evaluate the electrical properties of PEDOT:PSS-MP films. This evaluation is carried out after manufacturing simple devices in which the presence of the PEDOT:PSS improves the quality of the anode surface and facilitates the holes extraction in the device.

### 3.2. Electrical Characterization

**Figure 8:**
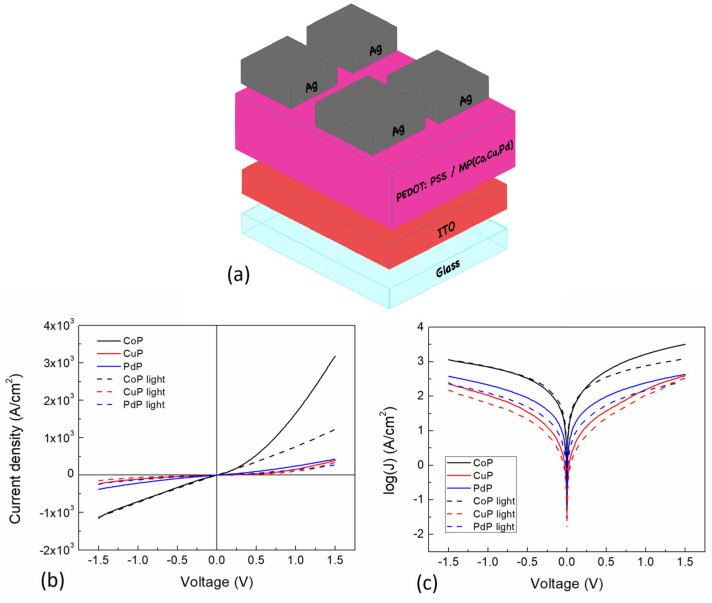
(**a**) Device structure for composite films. (**b**) J-V curves and its (**c**) semilogarithmic curve, for the different porphines under darkness and illuminated conditions.

Figure 8a shows the simple device structure of the PEDOT:PSS-MP composite films for the electrical characterization, where silver and ITO are used as cathode and anode, respectively. A conduction channel area of 1.39 × 10^−6^ cm^2^ was used. The PEDOT:PSS flexibility may be affected by the incorporation of each porphine as previously observed for other incorporated materials [53,54], whereas for the porphine no previous studies have been conducted. Figure 8b shows J-V curves and its (c) semilogarithmic curve for the different porphines under darkness and light conditions. As can be observed, the measured curves show a non-linear behavior that resembles a Schottky barrier device for all the MP films, where a non-symmetrical curve is observed for the forward and reverse bias regions. The latter indicates that the incorporation of the MPs into the PEDOT:PSS induces a junction formation, and the films current transport mechanisms are changed. Apparently these MPs are incorporated as nanoparticles within the PEDOT:PSS, affecting the electrical properties. In the forward bias region (Figure 8b), an increase in current density is observed for all the composite films curves in such a way that at 1.5 V it reaches 3.19 × 10^3^ A/cm^2^ for CoP (the highest value), 3.95 × 10^2^ A/cm^2^ for CuP, and 4.28 × 10^2^ A/cm^2^ for PdP. The value for CoP is eight times larger compared to the one obtained for CuP. These observation may be related to the CoP homogenous micro-grained morphology, denser film, less quinoid structure, and its lower interaction with the PEDOT:PSS, compared to the CuP composite film. These results suggest that the CuP composite film needle shaped structure morphology and the higher degree of interaction with the PEDOT:PSS quinoid structure affects the conduction mechanisms, diminishing the J-V characteristics. On the contrary, in the reverse bias region (Figure 8b), a decrease in all the composite films curves is observed. At 1.5 V it is observed that the CoP has the lowest current density of −1.16 × 10^3^ A/cm^2^, with the CuP of −2.49 × 10^2^ A/cm^2^ and the PdP of −3.82 × 10^2^ A/cm^2^. However, the CoP value is 4.6 times larger compared to the CuP. Moreover, J-V characteristic measurements were conducted under light conditions and plotted on Figure 8b,c for comparison with the J-V curves in darkness conditions. Figure 8b shows that CoP composite film presents, on the forward bias region at 1.5 V, a current density of 1.23 × 10^3^ A/cm^2^ (61% decrease), whereas the CuP and PdP of 3.37 × 10^2^ A/cm^2^ (15% decrease) and 2.69 × 10^2^ A/cm^2^ (37% decrease), respectively. This light induced change observations may be related to the change of the RMS and a more quinoid structure, which is higher for the CoP, affecting the light absorption and conductivity in a greater manner. On the reverse bias region at 1.5 V, there is a current density of −1.13 × 10^3^ A/cm^2^ for the CoP, and for the CuP and PdP of −1.53 × 10^2^ A/cm^2^ and −2.31 × 10^2^ A/cm^2^, respectively. These values correspond to a 3%, 39%, and 40% decrease, respectively for the CoP, CuP, and PdP composite films. Figure 8c allows a more detailed comparison between the semiconductor films and under light conditions, where a short circuit current (Jsc) variation can be observed. The latter is a consequence of the MP, by which the semiconductor material output could determine its application on optoelectronic devices, like solar cells, sensors, photodiodes, and others. In addition to the current density variations shown on Figure 8c, it is interesting to note that the reverse bias region curves for darkness conditions show a similar curve shape, contrary to what is observed for the forward bias region.

**Figure 9:**
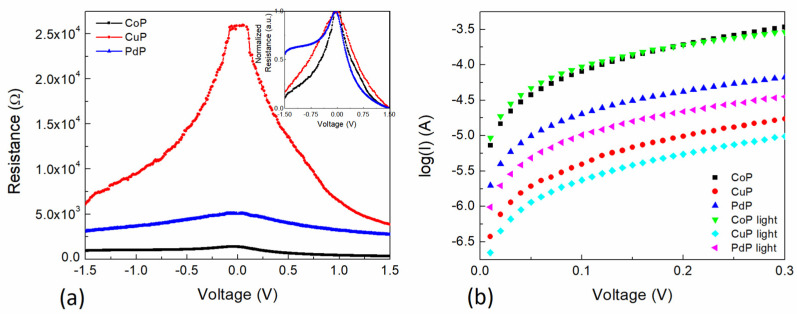
(**a**) Voltage dependent device resistance for the different porphines and (**b**) semilogarithmic I-V curves for the different porphines under darkness and illuminated conditions.

Figure 9a shows the voltage dependent semiconductor resistance for each of the compound films. As can be observed, the CuP film presents the highest resistance, and it approaches 2.60 × 10^4^ Ω at very low voltages. A lower resistance is presented by the PdP, which approaches 5.06 × 10^3^ Ω at very low voltages, and the lowest resistance is presented by the CoP, which approaches 1.37 × 10^3^ Ω at very low voltages. First, it is important to note that the semiconductor resistance decreases with the voltage increase, both negatively and positively. A more pronounced variation on the device resistance decrease is observed for the CuP composite film. In addition, a slight variation of the curve decay is observed between the forward and reverse bias for all the composite films. Figure 9a inset shows the normalized resistance to analyze the variation of the semiconductor resistance for the different MP composite films. It can be observed that all curves present different behavior. The PdP curve shows a very asymmetrical shape: for forward bias a steep decrease is observed, whereas for reverse bias a gentle decrease of almost the half of the previous observation is shown. It is interesting to note that for the forward bias region, the curves decay trend to zero at 1.5 V and the broadest curve is for the CuP, whereas the sharpest is for the PdP. However, for the reverse bias region, the curves decay to different values at −1.5 V, the CoP being the lowest, followed by CuP and PdP, respectively. The latter indicates that the PEDOT:PSS-MP composite films present different conduction mechanisms by changing the MP, and are bias dependent, which is a consequence of the charge carrier type mobility within the film. Figure 9b shows the semilogarithmic I-V curves for the different porphines under darkness and illuminated conditions for the 0–0.3 V voltage range. It can be observed a variation of almost 1.5 orders in magnitude for the current comparing the various composite films. Furthermore, under light conditions a more detailed effect can be observed. The CoP curve presents a higher current value for the light condition for voltages lower than 0.15 V, but for the CuP and the PdP the current values for the light condition are lower for all the voltages.

Table 3 shows the PEDOT:PSS-MP composite films’ electrical parameters. The conductivity was calculated from the slope of the IV curves; the Jsc (light), Vo (darkness), and Voc (light) were obtained from the intersection of the curves with the current and voltage axis; and the series resistance was calculated from the semilog IV curve. As can be seen in the table, the porphines have different electrical properties, for instance, the conductivity was calculated for the reverse and forward bias regions, decreasing from the cobalt porphine to the palladium porphine and to the copper porphine. The observed conductivity values yield between 1.23 × 10^2^ and 1.92 × 10^3^ Scm^−1^, being comparable to the doped PEDOT:PSS in previous observations [55,56], and it is improved for the CoP composite film. The improved conductivity might be related to an increase in the crystallinity and ordering of the PEDOT:PSS, the augment of the contact area, and the conductivity of the MPs. Furthermore, the phase segregation of PEDOT:PSS on PET increases its capability to transfer charges [57], supporting the previous observations. Nevertheless, the forward bias region presents larger conductivity values than the reverse bias region, showing an increment of 530 Scm^−1^ (38%), 35 Scm^−1^ (28%), and 42 Scm^−1^ (10%), for the CoP, CuP, and PdP, respectively. The latter indicates that despite the conductivity values for each MP, the lowest increment is observed for the PdP, suggesting that there is a strong interaction of the MPs with the surrounding PEDOT:PSS resulting in a variation of the electrical properties of the composite films. Table 3 also shows the Jo and Jsc for the different semiconductor films, related to Figure 9b observations. It is shown that PdP has the highest Jo (−1.38 × 10^−1^ A/cm^2^) and lowest Jsc (−4.13 × 10^−2^ A/cm^2^), whereas CuP has the lowest Jo (−2.54 × 10^−2^ A/cm^2^) and CoP has the highest Jsc (−3.92 × 10^−1^ A/cm^2^). In particular, for the Jsc, changing the MP may cause a variation of as much as 0.35 A/cm^2^. The latter indicates that there is an interesting current MP-dependent light effect and of ~0.16 A/cm^2^, ~0.01 A/cm^2^, and ~0.09 A/cm^2^, respectively, for the CoP, CuP, and PdP. In contrast, the open circuit voltage in darkness conditions (Vo) presents the highest voltage value for the PdP and the lowest for the CoP. For the open circuit voltage (Voc) it can be observed that the CuP presents the highest value of 5.98 × 10^−4^ V and PdP the lowest of 4.84 × 10^−4^ V, presenting a Voc variation of as much as 1.14 × 10^−4^ V, depending on the MP. Shunt and series resistance were obtained and presented in Table 3. The composite semiconductor films present a very low shunt resistance from 1.30 × 10^−3^ Ω to 1.58 × 10^−2^ Ω. In the case of the series resistance, it was calculated at 0.2 V for darkness and light conditions as shown in Table 3. The largest series resistance value is for the CuP, followed by the PdP and then by the CoP, which yield between 10^2^–10^4^ Ω, even for the light conditions. However, the series resistance values are higher for light conditions. For instance, the PdP composite film series resistance almost doubled, from 2.57 × 10^3^ Ω to 4.84 × 10^3^ Ω. Nevertheless, the largest shunt and series resistance values were obtained for the CuP, which may be caused by the morphological needle shaped structures observed previously and the high degree of interaction of the embedded CuP nanoparticles with the PEDOT:PSS.

## 4. Conclusions

Three composite films of poly(3,4-ethylenedioxythiophene):poly(styrenesulfonate) (PEDOT:PSS) and MPs (M = Co, Cu, Pd) have been prepared. The composite films were steam treated with IPA and the PEDOT was transformed to its quinoid structure. The optical parameters of PEDOT:PSS-MP films were characterized using spectrophotometric measurements, and at λ < 450 nm the films become transparent. At λ ≥ 450 nm, the absorbance in the films increases and the optical bandgap for these films is between 2.75 and 3.74 eV. However, the bandgap is within the interval required for semiconductor films. Electrical measurements show a non-linear behavior that resembles a Schottky barrier device for all the MP films, with a non-symmetrical curve. The CoP shows an 8 times larger current density compared to the CuP. A light induced change in the J-V curves is observed for all the composite films, and it is more pronounced for the CoP. The CuP presents the highest resistance of all the films: 10^3^–10^4^ Ω. The semiconductor resistance decreases with the voltage increase and its behavior depends on the MP, presenting different conduction mechanisms. The observed conductivity values yield between 1.23 × 10^2^ and 1.92 × 10^3^ Scm^−1^. The forward bias region presents larger conductivity values than the reverse bias region values. The MP may cause a Jsc and Voc variation of as much as 0.35 A/cm^2^ and 1.11 × 10^−4^ V. The largest shunt and series resistance were obtained for the CuP, which may be related to the morphology. Additionally, this film is the one that presents the best response when treated with IPA. Its bandgap is reduced after treatment to 2.75 eV and is the lowest bandgap compared to films with CoP and PdP. Thus, it is recommended to use the PEDOT:PSS-CoP on devices for high current density generation and photo-sensible applications, whereas the PEDOT:PSS-Cu is recommended high light absorption applications due to its bandgap.

## Figures and Tables

**Table 1:** Assignments of MPs in powder IR spectra.

CoP (cm^−1^)	CuP (cm^−1^)	PdP (cm^−1^)	Assignments *
3442	3438	3442	N–H _pyrrole_
2929, 2858	2929, 2856	2929, 2856	ν C–H _Ph_
1484	1482	1484	ν C=C _Ph_
1457	1459	1457	ν C=N
1367	1367	1367	ν C–N
1228, 1153, 1056	1217, 1151, 1060	1229, 1151, 1058	δ C–H _Ph_
1020	1019	1017	ν C–N _pyrrole_
750, 701, 667	750, 700, 669	754, 692, 670	γ C–H _Ph_
558	556	560	γ (pyr. fold)
516	520	512	δ C–C
451, 409	451, 408	453, 415	ν M–N

* Vibrational modes: ν (stretching), δ (bending in the plane), γ (bending out of plane).

**Table 2:** RMS and bandgap for PEDOT:PSS-MP composite films.

Film	RMS (nm)	Bandgap No Treatment (eV)	Bandgap with IPA Treatment (eV)
PEDOT:PSS-CoP	107.99 ± 7.7	2.18 ± 0.2	2.87 ± 0.4
PEDOT:PSS-CuP	43.175 ± 5.3	2.98 ± 0.1	2.75 ± 0.3
PEDOT:PSS-PdP	6.82 ± 1.11	2.97 ± 0.3	3.74 ± 0.1

**Table 3:** PEDOT:PSS-metalloporphine composite films electrical parameters.

Porphine	σ	σ	Jo	Jsc	Vo	Voc	R_shunt_	R_series_	R_series_
	(Scm^−1^)	(Scm^−1^)	(A/cm^2^)	(A/cm^2^)	(V)	(V)	(Ω)	(Ω)	(Ω)
	Reverse	Forward						Darkness @0.2 V	Light @0.2 V
Co	1.39 × 10^3^	1.92 × 10^3^	−2.28 × 10^−1^	−3.92 × 10^−1^	3.92 × 10^−4^	5.28 × 10^−4^	1.30 × 10^−3^	5.40 × 10^2^	5.71 × 10^2^
Cu	1.23 × 10^2^	1.58 × 10^2^	−2.54 × 10^−^^2^	−1.50 × 10^−2^	6.01 × 10^−4^	5.98 × 10^−4^	1.58 × 10^−2^	1.05 × 10^4^	1.92 × 10^4^
Pd	4.00 × 10^2^	4.42 × 10^2^	−1.38 × 10^−^^1^	−4.13 × 10^−2^	7.92 × 10^−4^	4.84 × 10^−4^	5.65 × 10^−3^	2.57 × 10^3^	4.84 × 10^3^

## Data Availability

Data is contained within the article.
